# Molecular and Computational Studies Reveal That Per- and Polyfluoroalkyl Substances Can Impair Protamine–DNA Interaction, Potentially Inducing DNA Damage

**DOI:** 10.3390/biom15091279

**Published:** 2025-09-04

**Authors:** Federica Musella, Maria Grazia Guarnieri, Simona Amore, Luigi Montano, Francesco Bertola, Salvatore Micali, Francesco Paolo Busardò, Carmen Di Giovanni, Gennaro Lettieri, Marina Piscopo

**Affiliations:** 1 Department of Biology, University of Naples Federico II, 80126 Naples, Italyguarnierimariagrazia@libero.it (M.G.G.); simo.amore@studenti.unina.it (S.A.);; 2 Andrology Unit and Service of Lifestyle Medicine in UroAndrology, Local Health Authority (ASL) Salerno, Coordination Unit of the network for Environmental and Reproductive Health (EcoFoodFertility Project), Oliveto Citra Hospital, 84020 Salerno, Italy; 3 ISDE—Medici per l’Ambiente, Sezione di Vicenza, 36100 Vicenza, Italy; 4 Department of Urology, University of Modena and Reggio Emilia, 41121 Modena, Italy; 5 Department of Biomedical Sciences and Public Health, Marche Polytechnic University, 60020 Ancona, Italy; 6 Department of Pharmacy, University of Naples Federico II, 80131 Naples, Italy; carmen.digiovanni75@gmail.com

**Keywords:** protamines-DNA interaction, DNA damage, molecular docking, PFAS, human spermatozoa

## Abstract

Interactions between protamines and DNA are essential for the correct structure of human sperm chromatin. Reproductive health can be adversely affected by environmental pollutants like per- and polyfluoroalkyl substances (PFAS). We previously reported that exposure to PFAS in the Veneto region causes alterations in sperm nuclear basic proteins (SNBP), along with reduced seminal antioxidant activity and increased lipoperoxides. This study analysed the protamine-to-histone ratio in SNBP and quantified the extent of DNA damage induced by SNBP in subjects in Veneto with serum perfluorooctanoic acid (PFOA) levels above the reference threshold. We found that all individuals with serum PFOA above the threshold exhibited grade three DNA damage, regardless of the protamine–histone ratio, which was generally altered but consistently shifted toward protamines. This indicate that exposure to PFAS can alter the protamine–histone ratio in these subjects. Moreover, SNBPs from these individuals showed reduced DNA-protective capacity under pro-oxidant conditions, suggesting a role in oxidative damage. To rationalize these effects, in this cross sectional study, we investigated the potential interactions between PFAS and human protamines by molecular docking analyses which showed that PFAS can form stable complexes with DNA through hydrophobic and polar interactions, especially with thymine pyrimidine rings. Further, docking analyses revealed that fluorine atoms in PFAS may interact with guanidinium groups in protamine P1 via electrostatic and van der Waals forces, competing with DNA for binding sites and potentially disrupting chromatin organisation. A ternary PFAS–DNA–protamine adduct may underpin the observed DNA damage. These results suggest that PFAS induce oxidative stress, which could affect male fertility.

## 1. Introduction

Sperm cells undergo a dramatic change in structure during spermatogenesis. The proteins that typically organise DNA in other cells, histones, are largely replaced by protamines [1]. Human sperm chromatin requires protamines to ensure proper packaging and protection of DNA. These smaller, highly basic proteins, replace almost all the histones, and form a very condensed and stable chromatin structure by tightly binding to and compacting the DNA [2,3,4,5,6,7,8,9]. This process is vital for protecting the sperm’s DNA from harm during its journey to the egg and for making sure that the genetic material is delivered correctly. Changes in protamine levels or their ability to bind to and compact DNA properly can lead to sperm DNA fragmentation because this can increase the vulnerability of sperm DNA to damage from various sources, such as free radicals and nucleases. All this can impact male fertility and potentially contribute to reproductive issues [10,11,12,13]. Reduced fertilisation rates are linked to sperm DNA damage, which may be due to impaired sperm function or the damaged DNA’s inability to integrate properly with the egg’s DNA. Attention is currently focusing increasingly on the extent and type of sperm DNA damage, which appears to predict fertilisation. Although the oocyte can repair double-strand breaks (DSBs) more easily than single-strand breaks (SSBs) in paternal DNA [14,15], the persistence of DSBs during fertilisation may cause genetic alterations and interruptions in embryonic development [16]. A number of environmental pollutants have been shown to induce alterations in the structure and function of sperm nuclear basic proteins (SNBP) [17,18,19,20,21,22,23,24], which may result in DNA damage and compromised fertility. Such alterations have the capacity to influence the manner in which SNBPs bind to DNA, thereby potentially disturbing the normal development and function of spermatozoa [25,26,27,28,29]. Among pollutants, per- and polyfluoroalkyl substances (PFASs) are persistent environmental contaminants [30,31,32,33,34]. These substances are environmental contaminants that are found in human tissues and in the environment. They pose a significant risk to reproductive health. Since the discovery of PFAS contamination in drinking water around the world, scientists have studied the health effects. These studies showed that PFAS causes oxidative stress and links to infertility and diseases [35,36,37,38,39,40,41]. In spring 2013, Veneto’s contaminated groundwater had PFAS from a late 1960s plant. A National Research Council (CNR)-Ministry of the Environment study revealed PFAS contamination of groundwater, surface water, and drinking water in the provinces of Vicenza, Padova, and Verona until autumn 2013. The 12 types of PFAS were traced back to a Trissino production plant [42]. The Veneto Regional Agency for Environmental Prevention and Protection investigated and found PFAS. A total of 130,000 people were exposed to these compounds as a result of the groundwater being contaminated. It was estimated by ARPA (Regional Environmental Protection Agency) in 1980 that groundwater contamination had reached the public aqueducts serving municipalities in the provinces of Vicenza, Verona and Padova. It was found in a 2016 study that serum levels of PFAS were higher in people living in areas with high levels of PFAS than in those living in areas with low levels [43]. The concentrations of perfluorooctanesulfonic acid (PFOS) and perfluorooctanoic acid (PFOA) were found to be lower in women than in men, and this has been linked to poorer reproductive health [44,45]. Sperm quality has been found to be negatively impacted by pollution, resulting in changes to standard sperm parameters. Interestingly, men can be infertile without any change being evident in their semen analyses. Otherwise, if an abnormal semen analysis is identified but no clear cause of these anomalies can be found, it is referred to as idiopathic infertility. A significant cause of idiopathic infertility arises from the excessive production of oxidative stress, with up to 80% of cases having elevated reactive oxygen species (ROS) [46,47,48,49]. We have previously demonstrated that the Veneto (VNT) semen samples exhibited higher levels of lipoperoxides and lower antioxidant activity than the samples from the Valley of Sele (VSL) area in the Campania Region, which is not polluted by PFAS [50]. In this study, we also demonstrated that the distribution of DNA damage types following SNBP addition under pro-oxidative conditions differed significantly between the two groups. SNBP in VNT subjects demonstrated a reduced capacity to protect DNA from oxidative damage. The results of this study suggested that exposure to PFAS produces oxidative stress in the semen of VNT [50]. In the present study, we analysed a larger set of samples of Veneto subjects presenting blood serum perfluorooctanoic acid (PFOA) levels above the threshold. In these subjects we analyzed the DNA damage grade and correlated these data with protamination levels and perfluorooctanoic acid (PFOA) blood serum levels. Our aim was to demonstrate the possibility of interactions between PFAS and protamines, with the ultimate goal of defining the molecular mechanisms by which PFAS causes DNA damage. To investigate this, we also employed molecular modelling.

## 2. Materials and Methods

### 2.1. Ethical Statement

The study was carried out in compliance with the guidelines and regulations that have been outlined. This is in accordance with the Code of Ethics of the World Medical Association. This is also in accordance with the Declaration of Helsinki. It is also in accordance with the definition of falls. within the scope of the EcoFoodFertility project (https://www.ecofoodfertility.it; accessed on 23 June 2025). The study protocol was approved by the Ethics Committee for Clinical Research. On 8 November 2019, the province of Vicenza held trials (prot. 113421) and made some changes. Approved once more on 29 July 2021 (Prot. 79483).

### 2.2. Recruitment

A planned preliminary evaluation of a data subset was included in this cross sectional study. This analysis was designed to produce broader results earlier in the study rather than waiting for all analytical determinations from the entire sample. This preliminary analysis used data from 507 young adults (18–35 years old) enrolled between 2022 and 2023 and living in municipalities in the red area (provinces of Padova, Verona and Vicenza). The Veneto Region’s DGR 2133/2016 and 691/2018 defined the municipalities supplied with polluted drinking water. The decision was made to proceed with registration in all municipalities within the commune affected by the supply of contaminated drinking water. For the purposes of this study, these municipalities were as follows: Albaredo d’Adige, Alonte, Arcole, Asigliano, Bevilacqua, Bosco S. Anna, Brendola, Cologna Veneta, Legnago, Lonigo, Minerbe, Montagnana, Noventa Vicentina, Orgiano, Pojana Maggiore, Pressana, Roveredo, Sarego, Terrazzo, Urbana, Veronella and Zimella. Specifically, the study analysed the semen of 99 men aged 18–35 (born after 1985) who lived in one of the municipalities in the provinces of Vicenza, Padua and Verona (VNT) for at least five years between 1985 and 2017, and whose mothers were pregnant or breastfeeding in the same area during the same period. The semen of these subjects was compared with that of 50 men of the same age from the Sele Valley (VSL), who were used as a control area. The latter was a low environmental impact area (https://www.arpacampania.it/; Accessed on 29 July 2025). There was no known illegal dumping of toxic waste, and the economy was based mainly on low- to medium-scale agriculture. economy based mainly on low- to medium-scale agriculture. Based on the pollutant concentrations detected in the area, the Sele Valley was categorised as a control area [50].

### 2.3. Inclusion, Exclusion and Confounding Criteria

The study’s inclusion criteria required participants to have a birth year of 1985 or later, a timeframe established based on assessments of when contaminated groundwater reached the municipal water supply. A further requirement was that participants must have been born in or have resided for at least five years in one of the 23 municipalities defined by the Veneto region as a “red zone” due to confirmed PFAS water pollution. Enrollment was voluntary, managed through a digital platform, and contingent upon the completion of a mandatory digital questionnaire exploring the residential history of the subject and their mother. Exclusion criteria were applied to any individual born and residing outside the red zone, those under the age of 18 or over the age of 35, and individuals with genetic syndromes such as Klinefelter or Down syndrome. Compliance with all criteria was verified through the questionnaire and a subsequent interview conducted by the International Society Doctors for the Environment (ISDE) before clinical analyses commenced. Finally, several clinical and pharmacological situations were evaluated as confounding variables, ascertained via testicular ultrasound and specific urological examinations for all participants. These included conditions like varicocele, orchitis, urethritis, prostatitis, and epididymitis. Also considered were severe systemic diseases and their associated treatments known to affect fertility, such as type II diabetes, neoplastic diseases, and the use of drugs that interfere with spermatogenesis (e.g., antidepressants, antibiotics, corticosteroids), sex hormone therapies, or chemo/radiation therapy.

### 2.4. PFAS’s Determination

For the analytical procedure, a 200 µL aliquot of the sample was fortified with an internal standard, followed by the addition of 600 µL of acetonitrile to facilitate protein precipitation. After vortexing and centrifugation at 4000× *g* for 10 min, the resulting supernatant was collected and transferred to a polypropylene tube containing 200 mg of a QuEChERS salt mixture (4:1 *w*/*w*, MgSO_4_:NaCl). The sample was again vortexed and centrifuged. The subsequent supernatant was isolated, transferred to a new polypropylene tube, and evaporated to dryness under a nitrogen stream. The residue was then reconstituted in 100 µL of a water–methanol solution (80:20, *v*/*v*) and transferred into polypropylene autosampler vials. A 10 µL volume of the prepared sample was injected into the UPLC-MS/MS system. The chromatographic separation of analytes was performed using an ACQUITY UPLC BEH C18 column (2.1 mm × 100 mm, 1.7 µm; Waters Corporation, Milford, MA, USA), with the column temperature maintained at 35 °C. A gradient elution was employed, using two mobile phases: mobile phase A, consisting of 2 mM ammonium acetate in a 95:5 water–methanol mixture, and mobile phase B, composed of 2 mM ammonium acetate in methanol. The mass spectrometer was equipped with an electrospray source operating in negative ionization mode (ESI-) and was set to multiple reaction monitoring (MRM) mode, acquiring two transitions for each analyte where possible.

### 2.5. Extraction of Sperm Nuclear Basic Proteins (SNBP) from Spermatozoa

Spermatozoa were isolated from semen samples by centrifugation at 5500× *g* for 30 min at 4 °C. The subsequent extraction of SNBP was performed using a modified procedure adapted from previously established methodologies [21]. In brief, the pellets were washed with solution A, composed: 20 mM Tris-HCl pH 8, 2 mM MgCl_2_, 0.5% Triton X-100. Following this wash the pellets were resuspended in 200 μL of phenylmethylsulfonyl fluoride (PMSF) 1 mM and then centrifuge at 8940× *g* for 5 min at 4 °C. The supernatant was discarded and the pellets were resuspended in solution B, which was composed of the following: 1 mM PMSF, 20 mM EDTA pH 8, 100 mM Tris-HCl pH 8. An equal volume of solution C was added: DTT 575 mM and reached 1 mL of volume with guanidine chloruro 6 M. The sample was mixed for 20 s. Then five volumes of cold ethanol 100% were added and incubated at −20 °C for 1 h. Subsequently, the samples were centrifuged, supernatant was discarded and 500 µL of 0.5 M HCl was added to the pellet to solubilize the SNBP. The samples were first placed in the thermoblock at 37 °C for 5 min, then vortexed for 20 sec, and incubated again at 37 °C for 2 min. Next, the samples were centrifuged at 17,530× *g* for 10 min at 4 °C. The supernatant was removed and transferred to tubes containing 125 µL of 100% trichloroacetic acid (TCA), intended to precipitate the basic proteins present in the suspension. Samples were incubated at 4 °C for 10 min, shaking them every 2 min to promote precipitation, and centrifuged at 17,530 × *g* for 10 min at 4 °C. The supernatant was discarded, and the resulting pellet was resuspended in 500 µL of a solution consisting of: 1% β-mercaptoethanol in acetone 100%. After centrifugation at 17,530 × *g* for 5 min at 4 °C, the pellet was washed a second time using the same solution and centrifugation conditions. In conclusion the β-mercaptoethanol and acetone were removed in vacuum centrifuge and resuspended in 55 μL of distilled water.

### 2.6. Electrophoresis of SNBP in AU-PAGE

The SNBPs extracted from spermatozoa were analysed using acetic acid-urea polyacrylamide gel electrophoresis (AUPAGE). AU-PAGE was performed as previously described [51,52], using a 15% (*w*/*v*) acrylamide solution (acrylamide–bisacrylamide ratio of 30:0.2). The gel, with a final volume of 8 mL, consists of 4 mL of acrylamide/N,N′-methylenebisacrylamide, 8 M urea, 5% acetic acid, 100 µL of TEMED and 140 µL of 10% APS. After polymerization, a pre-run of approximately 1 h was carried out at a constant voltage of 150 V using 5% acetic acid as a running buffer. A solution containing 4 µg of protein and 20 µL of a 20% M β-mercaptoethanol 100% and 8 M urea solution was then loaded onto the gel. Following this, 2 µL of 100% acetic acid and 2 µL of 0.001% pyronin were added after 1 h. The electrophoresis was carried out at a voltage of 100 V for approximately 1 h. Following this, the gels were then stained with Coomassie Blue Brilliant R-250 in accordance with the previously described method. Finally, the image was acquired using a Biorad GelDoc (ver. 6.0.1 build 34).

### 2.7. Plasmid DNA Extraction

The pGEX-2TK plasmid (4969 bp) from *E. coli* HB 101 cells was extracted using the ZymoPURETM Plasmid Miniprep Kit (Zymo Research Europe, Breisgau, Germany). The extraction process was carried out in strict accordance with the manufacturer’s guidelines, with only minor adjustments made to the temperature setting as outlined in Carbone et al. 2012 [53]. The plasmid was quantified using a NanoDrop (Thermo Fisher Scientific, Waltham, MA, USA) and analysed using 1% agarose gels.

### 2.8. DNA Protection Assay

The ability to protect the DNA from oxidative damage by SNBP was tested. The DNA oxidative damage was induced in the presence of 10 μM H_2_O_2_ and 5 μM CuCl_2_, which creates conditions conducive to the Fenton’s reaction. An increasing amount of SNBP was added to fix DNA 150 ng using protein/DNA weight/weight (*w*/*w*) ratios ranging from 0.4 to 0.8. To induce oxidative damage, the mixture was incubated at 37 °C for 30 min after H_2_O_2_ and CuCl_2_ were added. This addition occurred following a 5 min interaction between the protein and DNA at room temperature. Analysis was conducted on a 1% agarose gel, run at 100 V for 30 min in 1X TEB. DNA was stained using SafeView™ Classic (abm, Richmond, BC, Canada). Gel images were acquired with a Bio-Rad GelDoc (Hercules, CA, USA). Comprehensive information regarding DNA damage types can be found in the Supplementary Materials.

### 2.9. Computational Approach for Biomolecular Modeling

Modeling studies were performed using the MzDOCK pipeline [54]. Three-dimensional conformers of PFOA (Perfluorooctanoic acid) and PFAS (Perfluorooctanesulfonic acid) were retrieved from the PubChem database, prepared, and subjected to energy minimization using the MMFF94 force field. The tertiary structure of the biomolecule, protamine P1, was predicted using AlphaFold 2, a state-of-the-art AI-based tool for accurate protein structure prediction. The amino acid sequence of protamine P1 was obtained from UniProt [ID: P04553]. Hydrogen atoms were added to the biomolecule, and the protonation states of residues were adjusted to reflect physiological pH. The structure was further optimized through energy minimization using 250 steps of the steepest descent algorithm and conjugate gradient with the MMFF94 as force field. Molecular docking simulations were conducted using the Smina algorithm based on Autodock Vina. For each ligand (PFOA and PFAS), 50 top-ranked binding poses were generated (as defined by the “mode” parameter). The results included .pdbqt files of the final docked complexes and corresponding .log files containing docking scores (Vina score) expressed as affinity in kcal/mol. The docking grid was centered on the poly-arginine (poly-Arg) anchoring domain of protamine P1, based on structural data from Brewer et al., 2003 [55]. Molecular interactions between the ligands and the binding site residues of the biomolecule were analyzed using the PLIP (Protein–Ligand Interaction Profiler) tool [56] and visualized in PyMOL (The PyMOL Molecular Graphics System, Version 1.2r3pre, Schrödinger, LLC, New York, USA). Molecular modeling studies involving DNA–PFAS interactions were carried out using the HADDOCK platform for molecular docking simulations. Prior to docking, the DNA structure was energy minimized using the Universal Force Field (UFF) with 250 steps of steepest descent followed by 750 steps of conjugate gradient minimization to ensure a relaxed starting conformation. The HADDOCK protocol follows a three-step docking approach:

1. Rigid-body docking, involving randomization of ligand orientations and energy minimization (1000 structures generated);
2. Semi-flexible refinement, in which the interface regions are treated as flexible and refined using simulated annealing in torsion angle space (200 structures generated);
3. Final refinement, consisting of energy minimization in an explicit solvent model (200 structures generated).

Docked complexes were clustered based on root-mean-square deviation (RMSD) and number of common interfacial contacts. Each structure was scored using HADDOCK’s scoring function (HS), which incorporates several energetic components: intermolecular electrostatic energy (E_elec) and van der Waals energy (E_vdW), both calculated using the OPLS force field; an empirical desolvation energy term (E_desolv); buried surface area (E_BSA); and ambiguous interaction restraint energy (E_air). All figures were refined using the Canva design platform (https://www.canva.com/accessed 27 July 2025).

## 3. Results

**Figure 1:**
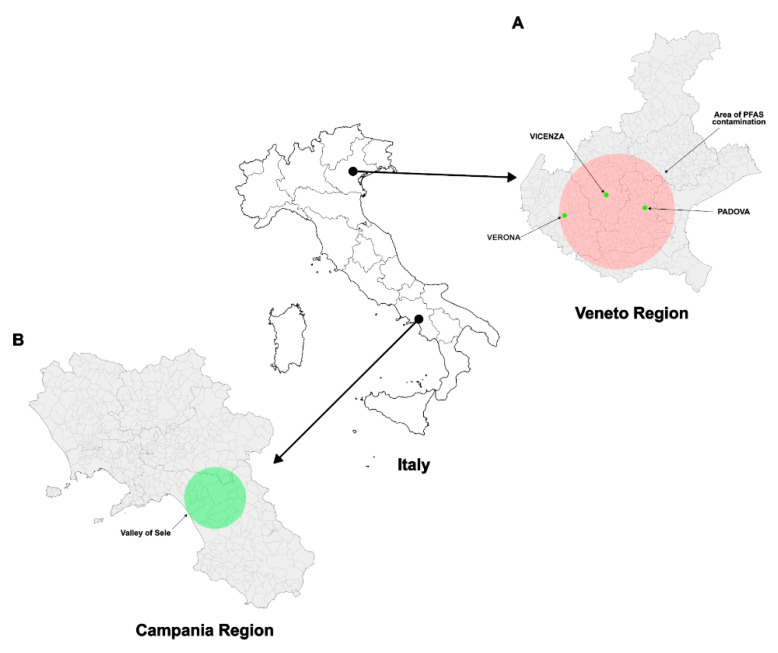
Map of recruitment’s areas: the Veneto region (Italy), highlighting the PFAS-contaminated area identified in 2013 due to wastewater discharges from a former chemical plant in Trissino (Vicenza). The contamination of groundwater and surface waters, distributed via public aqueducts, exposed approximately 140,000 residents. Semen samples were collected from young men across the provinces of Vicenza, Padova and Verona (**A**). The semen of these subjects was compared to that of subjects living in the Sele Valley (VSL), not contaminated to PFAS, in Campania region (**B**). Figure 1 reused from our work: [50].

In this study, we analysed the semen samples of 99 subjects residing in the Veneto region (Figure 1) who were exposed to perfluoroalkyl substances (PFAS) and showed blood serum perfluorooctanoic acid (PFOA) levels above the threshold.

**Figure 2:**
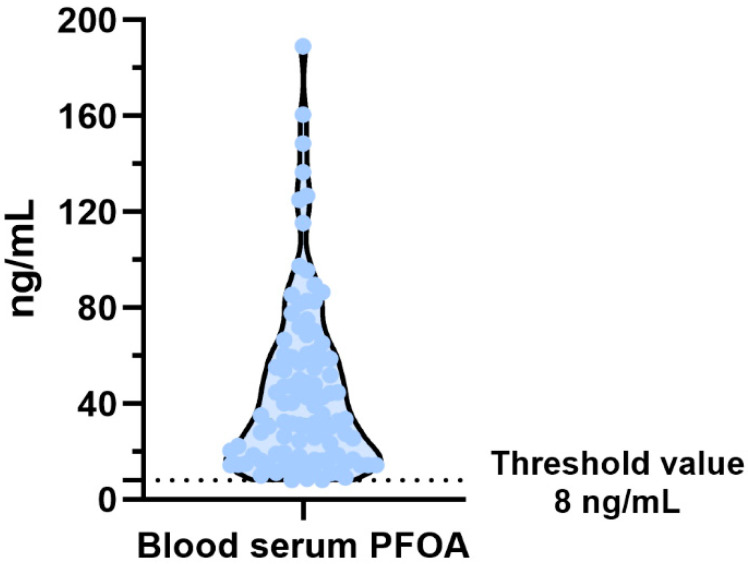
Violin plot of PFOA in the blood serum of subjects.

We analysed the accumulation of PFOA in the blood serum of these subjects and selected the 99 subjects with a PFOA value greater than the threshold limit of 8 ng/mL, as shown in Figure 2.

### 3.1. Analysis of the SNBP

**Figure 3:**
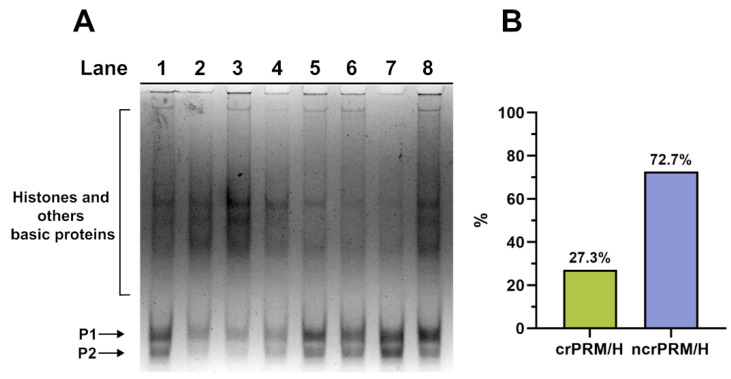
(**A**) Acid acetic polyacrylamide gel electrophoresis of spermatozoa samples in VNT subjects. The canonical protamine–histone ratio (CRPRM/H) is displayed in lanes 5, 6 and 7. Meanwhile, variegated cases of non-canonical ratios are demonstrated in lanes 1, 2, 3, 4 and 8. (**B**) Distribution of canonical and not canonical protamine–histone ratio. crPRM/H: canonical ratio Protamine–Histone; ncrRM/H: not canonical ratio Protamine–Histone. (Original image can be found in Supplementary Figure S1).

We extracted and analysed the SNBP from these subjects’ spermatozoa, finding a distribution of protamine–histone ratios (Figure 3) that was very different to that generally found in unpolluted areas (where the canonical ratio is 85% protamine and 15% histone for almost all samples), as reported in our previous work [19]. Figure 3 shows a representative gel, with lanes 5, 6 and 7 displaying the canonical protamine–histone ratio (crPRM/H), while lanes 1, 2, 3, 4 and 8 demonstrate variegated cases of non-canonical ratios but consistently shifted toward protamines. The percentage of the canonical protamine–histone (crPRM/H) to non-canonical (ncrPRM/H) ratio in these samples was 27.3 and 72.7, respectively.

### 3.2. Analysis of the Potential Involvment of SNBP in DNA Oxidative Damage

**Figure 4:**
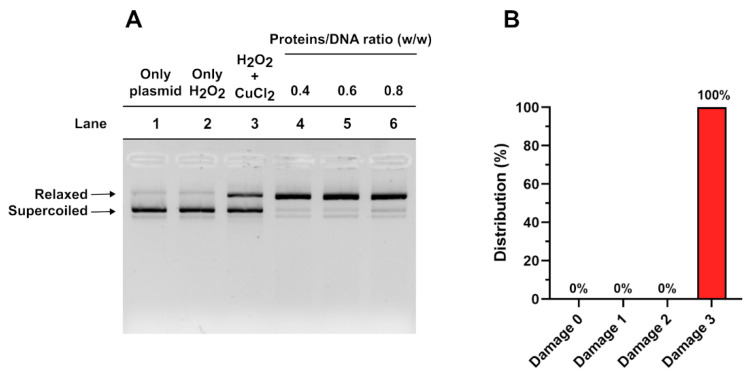
(**A**) DNA protection assays with SNBP (i.e., damage 3). (**B**) Type of DNA damage distribution. Lane1: only plasmid DNA; lane 2: plasmid DNA and H_2_O_2_, lane 3: plasmid DNA and H_2_O_2_ and CuCl_2_, lanes 4, 5 and 6: the same condition of lane 3 but with the adding of SNBP in the ratio 0.4, 0.6 and 0.8 with DNA, respectively. (Original image can be found in Supplementary Figure S3).

We also tested the SNBP for their ability to protect against or induce oxidative DNA damage in pro-oxidative conditions. As reported in our previous study, we categorized the DNA damage as grade 0 (no damage and protection of DNA from oxidative damage), grade 1, grade 2 and grade 3 (the maximum grade, i.e., the supercoiled form of plasmid DNA was completely converted to the relaxed form). As shown in the histograms in Figure 4B, we found that all subjects presented grade three DNA damage, which is illustrated by the agarose gel in Figure 4A. All of these subjects also exhibited a PFOA blood serum value higher than the threshold limit. No subjects exhibited damage grades 0, 1 or 2 (see Supplementary Figure S2), as indicated in Figure 4B.

Interestingly, damage of grade 3 was obtained from all SNBP samples, regardless of the protamine–histone ratio (Figure 3 and Figure 4).

### 3.3. Molecular Docking Analyses

**Figure 5:**
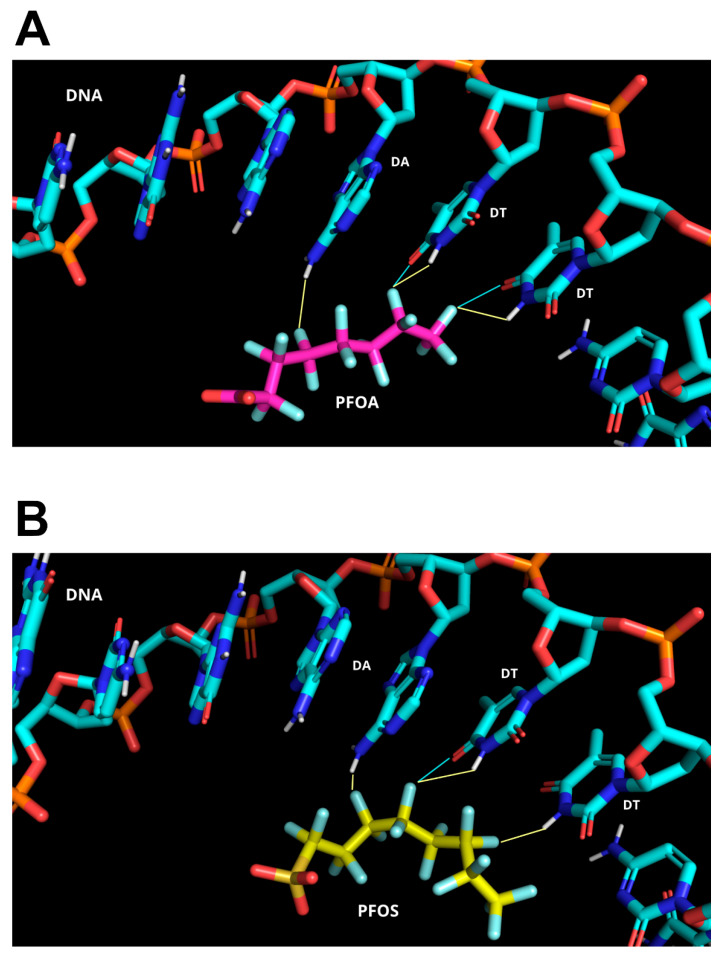
Predicted interactions between DNA and perfluoroalkyl substances (PFAS)F. (**A**) The most populated docking conformation of the PFOA–DNA complex. PFOA is shown as magenta sticks, while DNA is displayed as cyan sticks with atom-type coloring. (**B**) The most populated docking conformation of the PFOS–DNA complex. PFOS is visualized as yellow sticks, and DNA is shown in cyan sticks with atom-type coloring. H-bonding and halogen bonding interactions are illustrated with yellow and cyan lines, respectively.

The observation that damage of grade 3 was obtained from all SNBP samples, irrespective of the protamine–histone ratio, prompted us to undertake molecular docking analyses to evaluate whether a specific type of binding with protamines may occur with PFAS and thus result in DNA damage. Molecular modeling studies predict a strong binding affinity between PFAS and DNA. Molecular docking analyses indicate that the interaction is primarily driven by hydrophobic contacts and hydrogen bonding. Owing to the unique electronic properties of fluorine, capable of acting as both an electron donor and acceptor, PFAS can engage in electrostatic interactions, short hydrogen bonds, and halogen bonds, which further stabilize the complex. In the top-ranked solution predicted by HADDOCK, within the most populated cluster (HADDOCK score = −35.28; cluster size = 30), PFOA participates in short hydrogen bonds and halogen bonds with the purine nitrogen atoms of adenine, as well as with the carbonyl group and ring nitrogen within the pyrimidine structure of thymine. Further stabilization is provided by hydrophobic and van der Waals interactions between the alkyl chain of PFOA and the DNA backbone, as illustrated in Figure 5A,B. A similar binding pattern is observed in the top-ranked pose predicted by HADDOCK for PFOS (HADDOCK score = −28.76; cluster size = 20), characterized by a predominance of hydrogen bonds over halogen bonds, which were more prominent in the PFOA binding mode. This shift in interaction profile is consistent with the slightly lower HADDOCK score observed for PFOS compared to PFOA, suggesting a modestly reduced binding affinity. Additionally, hydrophobic and van der Waals interactions appear to play a less significant role in stabilizing the PFOS–DNA complex relative to PFOA (Figure 5).

**Figure 6:**
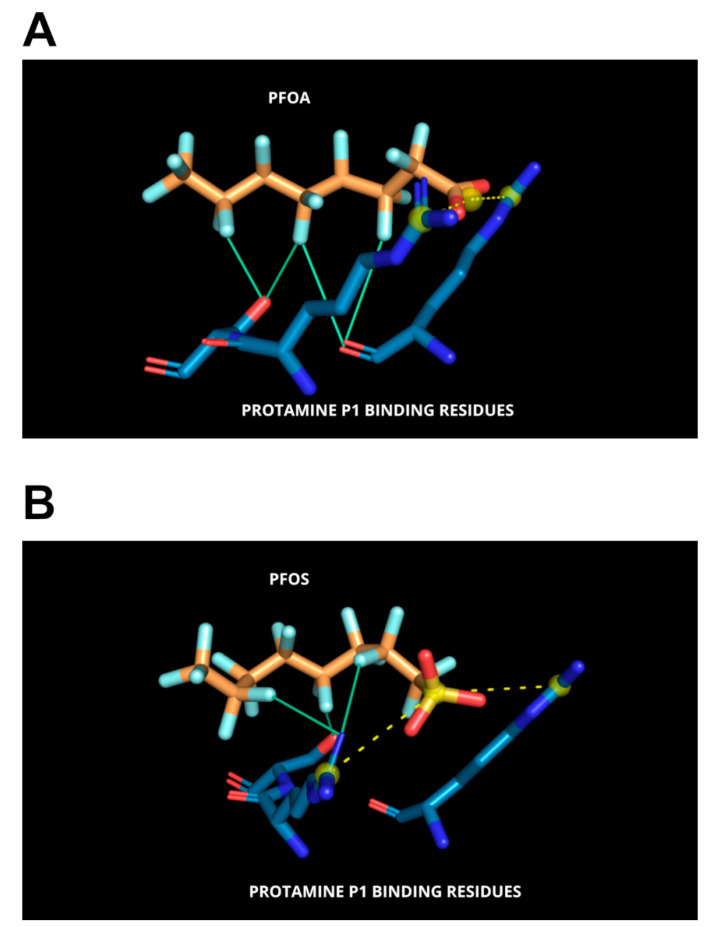
Predicted interactions between protamine P1 and perfluoroalkyl substances (PFAS). (**A**) Docked complex of PFOA with protamine P1 showing the highest binding affinity. For clarity, only the interacting protamine residues are displayed as blue sticks, colored by atom types. PFOA is shown as orange sticks, also colored by atom types. (**B**) Docked complex of PFOS with protamine P1. PFOS is represented as orange sticks, while the interacting protamine residues are shown as blue sticks, both colored by atom types. Bidentate salt bridges involving the guanidinium nitrogens of the arginine residues in the anchoring domain are represented by yellow dashed lines, while halogen bonds are depicted as cyan lines.

Our molecular docking studies involving protamine P1 and PFAS demonstrate that the polar head groups (carboxylic or sulfonic acids) of these perfluorinated compounds engage in stabilizing polar interactions with ionizable residues, most notably arginines, within the DNA-anchoring domain of P1. This interaction pattern closely mirrors that observed in PFAS–albumin complexes [57]. In the lowest-energy binding poses predicted by MzDOCK, the carboxylic group of PFOA (Vina score = −12.28 kcal/mol) and the sulfonic acid group of PFOS (Vina score = −10.52 kcal/mol) are tightly stabilized within the poly-Arg anchoring domain via bidentate salt bridges (Figure 6A,B).

**Figure 7:**
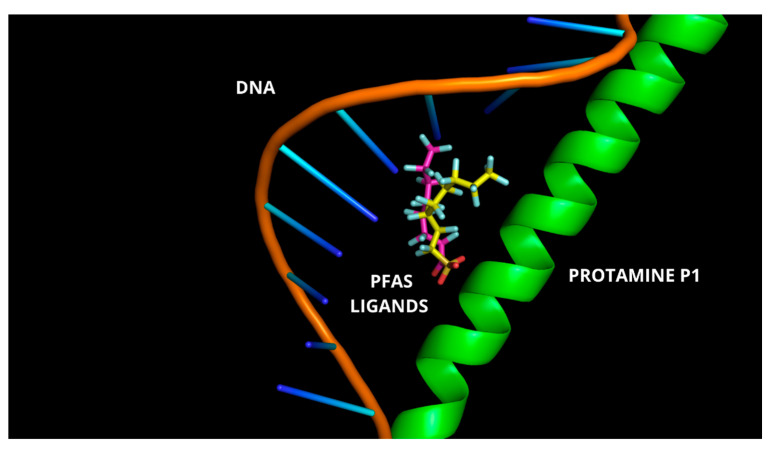
Best docked ternary complexes of DNA/protamine P1/PFAS. Protamine is illustrated as a magenta cartoon, DNA is displayed as sticks colored by atom type, and PFOA and PFOS are shown as magenta and yellow sticks, respectively, with atom-type coloring.

Furthermore, the polarizable fluorine atoms of these compounds are capable of forming halogen bonds with backbone oxygen or nitrogen atoms within the arginine-rich cluster, contributing additional stabilization to the complex. However, it cannot be ruled out that PFAS may also act as intercalating agents, forming stable adducts with both protamines and DNA (Figure 7) an aspect currently under investigation.

## 4. Discussion

It is well known that exposure to certain environmental and occupational chemicals may have an adverse effect on male fertility causing alterations in the interactions between sperm nuclear basic proteins and DNA. Pollutants can sometimes also produce epigenetic changes in these proteins [11] or in DNA and the effects of epigenetic alterations on several biological processes are well known [58,59,60]. There has been a significant increase in reports of the environmental and human health impacts of per- and polyfluoroalkyl substances (PFAS) in the peer-reviewed literature [61]. PFAS are ubiquitous and accumulate in the environment and the human body due to their stability and persistence. Epidemiological studies have revealed links between exposure to certain PFAS and various health issues, including impaired immune and thyroid function, liver disease, lipid and insulin imbalances, kidney disease, adverse reproductive and developmental outcomes, and cancer [62]. Currently, much of the available toxicity data for PFAS relates to a small number of chemicals, primarily legacy PFAS such as perfluorooctanoic acid and perfluorooctane sulfonate [63,64]. The impact of PFAS on male reproductive function is not well understood, as only a few studies have examined the link between PFAS exposure and male infertility, and the results have been inconsistent [65,66]. No correlation was found between PFAS exposure and semen fluid volume, sperm concentration, and total sperm count [67]. By contrast, other studies have demonstrated a negative correlation between elevated PFOA levels and certain conventional sperm parameters [66]. The goals of the present paper are to assess the state of the science regarding the effects of PFAS on the reproductive health of humans, with a particular focus on the potential interaction of PFAS with protamines and, in turn, with DNA oxidative damage. In this study, we analysed semen samples from 99 subjects residing in the Veneto region who had been exposed to PFAS and had blood serum PFOA levels above the threshold. Analysis of the SNBP in these subjects revealed an altered canonical protamine–histone (crPRM/H) to non-canonical (ncrPRM/H) ratio in the samples. Specifically, the ratio was canonical for 27.3% of the samples and non-canonical for 72.7%, which is significantly different to the ratio generally found in unpolluted areas, where the canonical ratio of 85% protamine and 15% histone is observed in almost all samples. The ability of the SNBP to protect against or induce oxidative DNA damage in pro-oxidative conditions was also tested. It was found that all subjects presented grade three DNA damage, with none exhibiting grades 0, 1 or 2. This observation gave rise to the hypothesis that a certain type of binding with protamines may occur with PFAS and thus cause DNA damage. This is because we have already demonstrated that some pollutants can interact with SNBP and produce DNA damage when these proteins bind DNA. We thus used molecular docking to verify this hypothesis. The literature reports that isothermal titration calorimetry and quantum chemical calculations have found that emerging perfluoroalkyl substances (PFASs) can bind to DNA bases (mainly thymine) via van der Waals forces and halogen bonds, with binding affinities ranging from 7.87 × 10^4^ to 6.54 × 10^6^ L/mol [68]. Consistent with these findings reported in the literature and supported by experimental data, molecular modeling studies predict a strong binding affinity between PFAS and DNA.

Indirect DNA conformational changes and potential damage may also result from the formation of stable complexes between PFAS and DNA-binding proteins such as protamines. Interactions between PFAS and a variety of biological targets have been extensively documented, including structural insights from X-ray crystallography [69]. Our molecular docking studies involving protamine P1 and PFAS show that the polar head groups (carboxylic or sulfonic acids) of these compounds form stabilising polar interactions with ionisable residues, particularly arginine residues, within the DNA-anchoring domain of P1. This highlights the importance of arginine residues in binding these proteins to DNA, in line with previous [70,71,72].

## 5. Conclusions

In conclusion, numerous epidemiological studies have established a link between environmental chemicals and male infertility. However, the precise mechanisms through which these chemicals affect fertility remain unclear. The aim of this study is to shed light on the effects of acute exposure to PFAS on human protamines. Our findings reveal that exposure to PFAS induces alterations in the protamine–histone ratio and renders these proteins capable of inducing oxidative damage to DNA, which could potentially compromise functions crucial for successful oocyte fertilisation. To the best of our knowledge, this is the first study to demonstrate specific interactions between PFAS and protamines. However, further research is required into the modes of action and adverse outcome pathways.

## 6. Limitation and Future Perspectives

The primary limitation of the present study is that the results are relating only to a portion of the total number of subjects recruited in the entire study. Consequently, a comprehensive analysis of a larger cohort will be conducted to validate these findings. The PFAS assessment is limited in scope, as it was conducted exclusively in blood serum for PFOA and PFOS, rather than in semen itself. In the future, we plan to analyse the presence of these contaminants also in the semen, in order to gain a clearer overview of the potential effects of PFAS on human male reproductive health. There are also significant differences in the toxicokinetic properties of PFAS. These must be taken into account to improve our understanding of their mechanism of action.

The research group is already conducting these analyses on a larger sample size. In addition, we will extend these investigations to female reproductive health. In particular, we are currently analyzing the effects of PFAS exposure on follicular fluid, aiming to clarify potential mechanisms of toxicity at the ovarian level.

## Data Availability

All data presented in this paper are available to request corresponding authors.

## Supplementary Materials

The following supporting information can be downloaded at https://www.mdpi.com/article/10.3390/biom15091279/s1: Supplementary Figure S1: Original image of Figure 3A. Supplementary Figure S2: Analysis on a 1% agarose gel of pGEM3 plasmid DNA damage induced by H_2_O_2_ in the presence of SNBPs. The different damage grade groups are grade 0 (**A**), grade 1 (**B**), grade 2 (**C**) and grade 3 (**D**). Supplementary Figure S3: Original image of Figure 4A.

## References

[1] Fioretti F.M., Febbraio F., Carbone A., Branno M., Carratore V., Fucci L., Ausió J., Piscopo M. (2012). A Sperm Nuclear Basic Protein from the Sperm of the Marine Worm *Chaetopterus variopedatus* with Sequence Similarity to the Arginine-Rich C-Termini of Chordate Protamine-Likes. DNA Cell Biol..

[2] Moritz L., Hammoud S.S. (2022). The Art of Packaging the Sperm Genome: Molecular and Structural Basis of the Histone-To-Protamine Exchange. Front. Endocrinol..

[3] Zini A., Phillips S., Courchesne A., Boman J.M., Baazeem A., Bissonnette F., Kadoch I.J., Gabriel M.S. (2009). Sperm Head Morphology Is Related to High Deoxyribonucleic Acid Stainability Assessed by Sperm Chromatin Structure Assay. Fertil. Steril..

[4] Cho C.-L., Agarwal A. (2017). Role of Sperm DNA Fragmentation in Male Factor Infertility: A Systematic Review. Arab. J. Urol..

[5] Zurera-Egea C., Mateo S., Novo S., Asensio M., Boada M., Antich M., Rovira S., Sarrate Z., Blanco J., Anton E. (2025). The Utility of Sperm DNA Fragmentation as a Diagnostic Tool for Male Infertility and Its Predictive Value for Assisted Reproductive Technology Outcomes. Int. J. Mol. Sci..

[6] de la Iglesia A., Jodar M., Oliva R., Castillo J. (2023). Insights into the Sperm Chromatin and Implications for Male Infertility from a Protein Perspective. WIREs Mech. Dis..

[7] Okada Y. (2022). Sperm Chromatin Structure: Insights from In Vitro to In Situ Experiments. Curr. Opin. Cell Biol..

[8] Lewis J.D., Saperas N., Song Y., Zamora M.J., Chiva M., Ausió J. (2004). Histone H1 and the Origin of Protamines. Proc. Natl. Acad. Sci. USA.

[9] Balhorn R. (2007). The Protamine Family of Sperm Nuclear Proteins. Genome Biol..

[10] Hærvig K.K., Petersen K.U., Hougaard K.S., Lindh C., Ramlau-Hansen C.H., Toft G., Giwercman A., Høyer B.B., Flachs E.M., Bonde J.P. (2022). Maternal Exposure to Per- and Polyfluoroalkyl Substances (PFAS) and Male Reproductive Function in Young Adulthood: Combined Exposure to Seven PFAS. Environ. Health Perspect.

[11] Abdulkadir A., Kandel S., Lewis N., D’Auvergne O., Rosby R., Hossain E. (2025). Epigenetic Consequences of In Utero PFAS Exposure: Implications for Development and Long-Term Health. Int. J. Environ. Res. Public Health.

[12] Agarwal A., Farkouh A., Parekh N., Zini A., Arafa M., Kandil H., Tadros N., Busetto G.M., Ambar R., Parekattil S. (2022). Sperm DNA Fragmentation: A Critical Assessment of Clinical Practice Guidelines. World J. Men’s Health.

[13] Siqueira M.H.B. (2025). Sperm DNA Fragmentation: A Narrative Review. Hum. Reprod. Arch..

[14] Ribas-Maynou J., García-Peiró A., Fernandez-Encinas A., Amengual M.J., Prada E., Cortés P., Navarro J., Benet J. (2012). Double Stranded Sperm DNA Breaks, Measured by Comet Assay, Are Associated with Unexplained Recurrent Miscarriage in Couples without a Female Factor. PLoS ONE.

[15] Ribas-Maynou J., Fernández-Encinas A., García-Peiró A., Prada E., Abad C., Amengual M.J., Navarro J., Benet J. (2014). Human Semen Cryopreservation: A Sperm DNA Fragmentation Study with Alkaline and Neutral Comet Assay. Andrology.

[16] Derijck A., van der Heijden G., Giele M., Philippens M., de Boer P. (2008). DNA Double-Strand Break Repair in Parental Chromatin of Mouse Zygotes, the First Cell Cycle as an Origin of de Novo Mutation. Hum. Mol. Genet..

[17] Nunzio A.D., Giarra A., Toscanesi M., Amoresano A., Piscopo M., Ceretti E., Zani C., Lorenzetti S., Trifuoggi M., Montano L. (2022). Comparison between Macro and Trace Element Concentrations in Human Semen and Blood Serum in Highly Polluted Areas in Italy. Int. J. Environ. Res. Public Health.

[18] Lettieri G., Carusone N., Notariale R., Prisco M., Ambrosino A., Perrella S., Manna C., Piscopo M. (2022). Morphological, Gene, and Hormonal Changes in Gonads and In-Creased Micrococcal Nuclease Accessibility of Sperm Chromatin Induced by Mercury. Biomolecules.

[19] Perrone P., Lettieri G., Marinaro C., Longo V., Capone S., Forleo A., Pappalardo S., Montano L., Piscopo M. (2022). Molecular Alterations and Severe Abnormalities in Spermatozoa of Young Men Living in the “Valley of Sacco River” (Latium, Italy): A Preliminary Study. Int. J. Environ. Res. Public Health.

[20] Lettieri G., Notariale R., Ambrosino A., Di Bonito A., Giarra A., Trifuoggi M., Manna C., Piscopo M. (2021). Spermatozoa Transcriptional Response and Alterations in PL Proteins Properties after Exposure of *Mytilus galloprovincialis* to Mercury. Int. J. Mol. Sci..

[21] Ferrero G., Festa R., Follia L., Lettieri G., Tarallo S., Notari T., Giarra A., Marinaro C., Pardini B., Marano A. (2024). Small Noncoding RNAs and Sperm Nuclear Basic Proteins Reflect the Environmental Impact on Germ Cells. Mol. Med..

[22] Lettieri G., Notariale R., Carusone N., Giarra A., Trifuoggi M., Manna C., Piscopo M. (2021). New Insights into Alterations in PL Proteins Affecting Their Binding to DNA after Exposure of *Mytilus galloprovincialis* to Mercury—A Possible Risk to Sperm Chromatin Structure?. Int. J. Mol. Sci..

[23] Marinaro C., Lettieri G., Chianese T., Bianchi A.R., Zarrelli A., Palatucci D., Scudiero R., Rosati L., De Maio A., Piscopo M. (2024). Exploring the Molecular and Toxicological Mechanism Associated with Interactions between Heavy Metals and the Reproductive System of Mytilus Galloprovincialis. Comp. Biochem. Physiol. C Toxicol. Pharmacol..

[24] Kumaresan A., Yadav P., Sinha M.K., Nag P., John Peter E.S.K., Mishra J.S., Kumar S. (2024). Male Infertility and Perfluoroalkyl and Poly-Fluoroalkyl Substances: Evidence for Alterations in Phosphorylation of Proteins and Fertility-Related Functional Attributes in Bull Spermatozoa†. Biol. Reprod..

[25] Sengupta P., Dutta S., Liew F.F., Dhawan V., Das B., Mottola F., Slama P., Rocco L., Roychoudhury S. (2023). Environmental and Genetic Traffic in the Journey from Sperm to Offspring. Biomolecules.

[26] Shi L., Tao L., Zong Y., Gao C., Han B., Gao T., Huang C., Liu B., Wu H., Tang D. (2025). Seminal Per- and Polyfluoroalkyl Substance Exposure and Sperm Quality Impairment: From Toxic Target to Rescue. Environ. Int..

[27] Tarapore P., Ouyang B. (2021). Perfluoroalkyl Chemicals and Male Reproductive Health: Do PFOA and PFOS Increase Risk for Male Infertility?. Int. J. Environ. Res. Public Health.

[28] Sun Z., Wen Y., Wang B., Deng S., Zhang F., Fu Z., Yuan Y., Zhang D. (2023). Toxic Effects of Per- and Polyfluoroalkyl Substances on Sperm: Epidemiological and Experimental Evidence. Front. Endocrinol..

[29] Alamo A., La Vignera S., Mogioì L.M., Crafa A., Barbagallo F., Cannarella R., Aversa A., Calogero A.E., Condorelli R.A. (2024). In-Vitro Effects of Perfluorooctanoic Acid on Human Sperm Function: What Are the Clinical Consequences?. J. Clin. Med..

[30] Leung S.C.E., Wanninayake D., Chen D., Nguyen N.-T., Li Q. (2023). Physicochemical Properties and Interactions of Perfluoroalkyl Substances (PFAS)—Challenges and Opportunities in Sensing and Remediation. Sci. Total. Environ..

[31] Panieri E., Baralic K., Djukic-Cosic D., Buha Djordjevic A., Saso L. (2022). PFAS Molecules: A Major Concern for the Human Health and the Environment. Toxics.

[32] Dams R., Ameduri B. (2025). Essential Per- and Polyfluoroalkyl Substances (PFAS) in Our Society of the Future. Molecules.

[33] Yang Y., Wang J., Tang S., Qiu J., Luo Y., Yang C., Lai X., Wang Q., Cao H. (2025). Per- and Polyfluoroalkyl Substances (PFAS) in Consumer Products: An Overview of the Occurrence, Migration, and Exposure Assessment. Molecules.

[34] Fischer F.C., Ludtke S., Thackray C., Pickard H.M., Haque F., Dassuncao C., Endo S., Schaider L., Sunderland E.M. (2024). Binding of Per- and Polyfluoroalkyl Substances (PFAS) to Serum Proteins: Implications for Toxicokinetics in Humans. Environ. Sci. Technol..

[35] Frisbee S.J., Brooks A.P., Maher A., Flensborg P., Arnold S., Fletcher T., Steenland K., Shankar A., Knox S.S., Pollard C. (2009). The C8 Health Project: Design, Methods, and Participants. Environ. Health Perspect..

[36] Agarwal A., Virk G., Ong C., du Plessis S.S. (2014). Effect of Oxidative Stress on Male Reproduction. World J. Men’s Health.

[37] Wielsøe M., Long M., Ghisari M., Bonefeld-Jørgensen E.C. (2015). Perfluoroalkylated Substances (PFAS) Affect Oxidative Stress Biomarkers In Vitro. Chemosphere.

[38] Wang W., Hong X., Zhao F., Wu J., Wang B. (2023). The Effects of Perfluoroalkyl and Polyfluoroalkyl Substances on Female Fertility: A Systematic Review and Meta-Analysis. Environ. Res..

[39] Specht I.O., Hougaard K.S., Spanò M., Bizzaro D., Manicardi G.C., Lindh C.H., Toft G., Jönsson B.A.G., Giwercman A., Bonde J.P.E. (2012). Sperm DNA Integrity in Relation to Exposure to Environmental Perfluoroalkyl Substances—A Study of Spouses of Pregnant Women in Three Geographical Regions. Reprod. Toxicol..

[40] Sun F., Lin Y., Pan A., Meng T.-Q., Xiong C.-L., Wang Y.-X., Liu X., Chen D. (2025). Per- and Polyfluoroalkyl Substances in Semen Associated with Repeated Measures of Semen Quality in Healthy Adult Men. Environ. Sci. Technol..

[41] Sciorio R., Greco P.F., Greco E., Tramontano L., Elshaer F.M., Fleming S. (2025). Potential Effects of Environmental Toxicants on Sperm Quality and Potential Risk for Fertility in Humans. Front. Endocrinol..

[42] Canova C., Barbieri G., Zare Jeddi M., Gion M., Fabricio A., Daprà F., Russo F., Fletcher T., Pitter G. (2020). Associations between Perfluoroalkyl Substances and Lipid Profile in a Highly Exposed Young Adult Population in the Veneto Region. Environ. Int..

[43] Ingelido A.M., Abballe A., Gemma S., Dellatte E., Iacovella N., De Angelis G., Zampaglioni F., Marra V., Miniero R., Valentini S. (2018). Biomonitoring of Perfluorinated Compounds in Adults Exposed to Contaminated Drinking Water in the Veneto Region, Italy. Environ. Int..

[44] Pitter G., Da Re F., Canova C., Barbieri G., Zare Jeddi M., Daprà F., Manea F., Zolin R., Bettega A.M., Stopazzolo G. (2020). Serum Levels of Perfluoroalkyl Substances (PFAS) in Adolescents and Young Adults Exposed to Contaminated Drinking Water in the Veneto Region, Italy: A Cross-Sectional Study Based on a Health Surveillance Program. Environ. Health Perspect..

[45] Rickard B.P., Rizvi I., Fenton S.E. (2022). Per- and Poly-Fluoroalkyl Substances (PFAS) and Female Reproductive Outcomes: PFAS Elimination, Endocrine-Mediated Effects, and Disease. Toxicology.

[46] Bui A.D., Sharma R., Henkel R., Agarwal A. (2018). Reactive Oxygen Species Impact on Sperm DNA and Its Role in Male Infertility. Andrologia.

[47] Sengupta P., Roychoudhury S., Nath M., Dutta S. (2022). Oxidative Stress and Idiopathic Male Infertility. Adv. Exp. Med. Biol..

[48] Aktan G., Doğru-Abbasoğlu S., Küçükgergin C., Kadıoğlu A., Özdemirler-Erata G., Koçak-Toker N. (2013). Mystery of Idiopathic Male Infertility: Is Oxidative Stress an Actual Risk?. Fertil. Steril..

[49] Saleh R.A., Agarwal A., Nada E.A., El-Tonsy M.H., Sharma R.K., Meyer A., Nelson D.R., Thomas A.J. (2003). Negative Effects of Increased Sperm DNA Damage in Relation to Seminal Oxidative Stress in Men with Idiopathic and Male Factor Infertility. Fertil. Steril..

[50] Marinaro C., Bianchi A.R., Guerretti V., Barricelli G., Berman B., Bertola F., Micali S., Busardò F.P., Di Giorgi A., De Maio A. (2025). Molecular Alterations in Semen of Per-And Polyfluoroalkyl Substance Exposed Subjects: Association Between DNA Integrity, Antioxidant Capacity and Lipoperoxides. Antioxidants.

[51] Piscopo M. (2019). Seasonal Dependence of Cadmium Molecular Effects on *Mytilus galloprovincialis* (Lamarck, 1819) Protamine-like Protein Properties. Mol. Reprod. Dev..

[52] Lettieri G., Maione M., Ranauda M.A., Mele E., Piscopo M. (2019). Molecular Effects on Spermatozoa of *Mytilus galloprovincialis* Exposed to Hyposaline Conditions. Mol. Reprod. Dev..

[53] Carbone A., Fioretti F.M., Fucci L., Ausió J., Piscopo M. (2012). High Efficiency Method to Obtain Supercoiled DNA with a Commercial Plasmid Purification Kit. Acta Biochim. Pol..

[54] Kabier M., Gambacorta N., Trisciuzzi D., Kumar S., Nicolotti O., Mathew B. (2024). MzDOCK: A Free Ready-to-Use GUI-Based Pipeline for Molecular Docking Simulations. J. Comput. Chem..

[55] Brewer L.R., Bianco P.R. (2008). Laminar Flow Cells for Single-Molecule Studies of DNA-Protein Interactions. Nat. Methods.

[56] Adasme M.F., Linnemann K.L., Bolz S.N., Kaiser F., Salentin S., Haupt V.J., Schroeder M. (2021). PLIP 2021: Expanding the Scope of the Protein–Ligand Interaction Profiler to DNA and RNA. Nucleic Acids Res..

[57] Crisalli A.M., Cai A., Cho B.P. (2023). Probing the Interactions of Perfluorocarboxylic Acids of Various Chain Lengths with Human Serum Albumin: Calorimetric and Spectroscopic Investigations. Chem. Res. Toxicol..

[58] Stoccoro A., Coppedè F. (2024). Exposure to Metals, Pesticides, and Air Pollutants: Focus on Resulting DNA Methylation Changes in Neurodegenerative Diseases. Biomolecules.

[59] Passaro D., Rana G., Piscopo M., Viggiano E., De Luca B., Fucci L. (2010). Epigenetic Chromatin Modifications in the Cortical Spreading Depression. Brain Res..

[60] Rana G., Donizetti A., Virelli G., Piscopo M., Viggiano E., De Luca B., Fucci L. (2012). Cortical Spreading Depression Differentially Affects Lysine Methylation of H3 Histone at Neuroprotective Genes and Retrotransposon Sequences. Brain Res..

[61] Dehghani M.H., Aghaei M., Bashardoust P., Rezvani Ghalhari M., Nayeri D., Malekpoor M., Sheikhi S., Shi Z. (2025). An Insight into the Environmental and Human Health Impacts of Per- and Polyfluoroalkyl Substances (PFAS): Exploring Exposure Pathways and Their Implications. Environ. Sci. Eur..

[62] Boston C., Keck S., Naperala A., Collins J. (2025). The Evolution of PFAS Epidemiology: New Scientific Developments Call into Question Alleged “Probable Links” between PFOA and Kidney Cancer and Thyroid Disease. Front. Public Health.

[63] Mahoney H., Xie Y., Brinkmann M., Giesy J.P. (2022). Next Generation Per- and Poly-Fluoroalkyl Substances: Status and Trends, Aquatic Toxicity, and Risk Assessment. Eco-Environ. Health.

[64] Fenton S.E., Ducatman A., Boobis A., DeWitt J.C., Lau C., Ng C., Smith J.S., Roberts S.M. (2021). Per- and Polyfluoroalkyl Substance Toxicity and Human Health Review: Current State of Knowledge and Strategies for Informing Future Research. Environ. Toxicol. Chem..

[65] Raymer J.H., Michael L.C., Studabaker W.B., Olsen G.W., Sloan C.S., Wilcosky T., Walmer D.K. (2012). Concentrations of Perfluorooctane Sulfonate (PFOS) and Perfluorooctanoate (PFOA) and Their Associations with Human Semen Quality Measurements. Reprod. Toxicol..

[66] Joensen U.N., Bossi R., Leffers H., Jensen A.A., Skakkebaek N.E., Jørgensen N. (2009). Do Perfluoroalkyl Compounds Impair Human Semen Quality?. Environ. Health Perspect..

[67] Vested A., Ramlau-Hansen C.H., Olsen S.F., Bonde J.P., Støvring H., Kristensen S.L., Halldorsson T.I., Rantakokko P., Kiviranta H., Ernst E.H. (2014). In Utero Exposure to Persistent Organochlorine Pollutants and Reproductive Health in the Human Male. Reproduction.

[68] Qin C., Xiang L., Wang Y.-Z., Yu P.-F., Meng C., Li Y.-W., Zhao H.-M., Hu X., Gao Y., Mo C.-H. (2024). Binding Interaction of Environmental DNA with Typical Emerging Perfluoroalkyl Acids and Its Impact on Bioavailability. Sci. Total. Environ..

[69] Zhao L., Teng M., Zhao X., Li Y., Sun J., Zhao W., Ruan Y., Leung K.M.Y., Wu F. (2023). Insight into the Binding Model of Per- and Polyfluoroalkyl Substances to Proteins and Membranes. Environ. Int..

[70] Piscopo M., Trifuoggi M., Scarano C., Gori C., Giarra A., Febbraio F. (2018). Relevance of Arginine Residues in Cu(II)-Induced DNA Breakage and Proteinase K Resistance of H1 Histones. Sci. Rep..

[71] Piscopo M., Conte M., Di Paola F., Conforti S., Rana G., De Petrocellis L., Fucci L., Geraci G. (2010). Relevance of Arginines in the Mode of Binding of H1 Histones to DNA. DNA Cell Biol..

[72] DeRouchey J., Hoover B., Rau D.C. (2013). A Comparison of DNA Compaction by Arginine and Lysine Peptides: A Physical Basis for Arginine Rich Protamines. Biochemistry.

