# Supplementary material for: Molecular and Computational Studies Reveal That Per- and Polyfluoroalkyl Substances Can Impair Protamine–DNA Interaction, Potentially Inducing DNA Damage
Source: Biomolecules. 2025 Sep 4;15(9):1279. doi: 10.3390/biom15091279 (PMC12467272; doi:10.3390/biom15091279)
Supplement: Supplementary file 1 [file biomolecules-15-01279-s001.zip › Supplementary materials.pdf]

Molecular and computational studies reveal that per- and polyfluoroalkyl substances can impair protamine–DNA interaction, potentially inducing DNA damage

Federica Musella[1,†], Maria Grazia Guarnieri[1,†], Simona Amore[1], Luigi Montano[2], Francesco Bertola[3], Salvatore Micali[4], Francesco Paolo Busardò[5], Carmen Di Giovanni[6], Gennaro Lettieri[1,‡] and Marina Piscopo[1,*,‡]

[1] Department of Biology, University of Naples Federico II, Naples 80126, Italy, federica.musella@unina.it (F.M.); guarnierimariagrazia@libero.it (M.G.G.); simo.amore@studenti.unina.it (S.A.); gennaro.lettieri@unina.it (G.L.); marina.piscopo@unina.it (M.P.)

[2] Andrology Unit and Service of Lifestyle Medicine in UroAndrology, Local Health Authority (ASL) Salerno, Coordination Unit of the network for Environmental and Reproductive Health (EcoFoodFertility project), Oliveto Citra Hospital, Salerno 84020, Italy; l.montano@aslsalerno.it (L.M.)

[3] ISDE—Medici per l'Ambiente, Sezione di Vicenza, 36100 Vicenza, Italy; franci.bertola@gmail.com (F.B.)

[4] Department of Urology, University of Modena and Reggio Emilia, 41121 Modena, Italy; salvatore.micali@unimore.it (S.M.)

[5] Department of Biomedical Sciences and Public Health, Marche Polytechnic University, 60020 Ancona, Italy; fra.busardo@gmail.com (F.P.B.)

[6] Department of Pharmacy, University of Naples Federico II, Naples 80131, Italy; carmen.digiovanni75@gmail.com (C.D.G.)

†These authors contributed equally to this work and are co-first
‡These authors are co-last
* Correspondence: marina.piscopo@unina.it (M.P.)

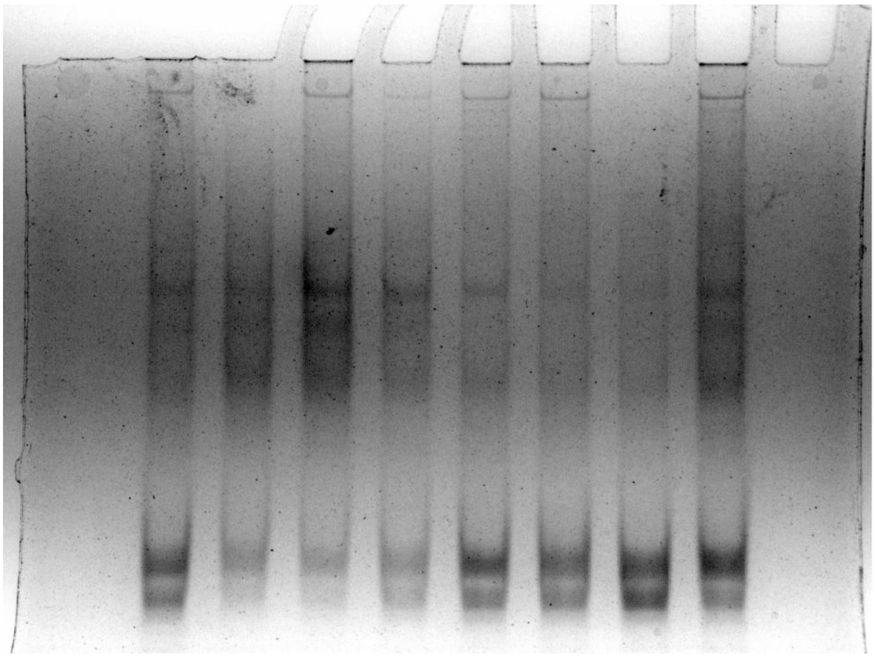

Supplementary Figure S1: Original image of Figure 3A.

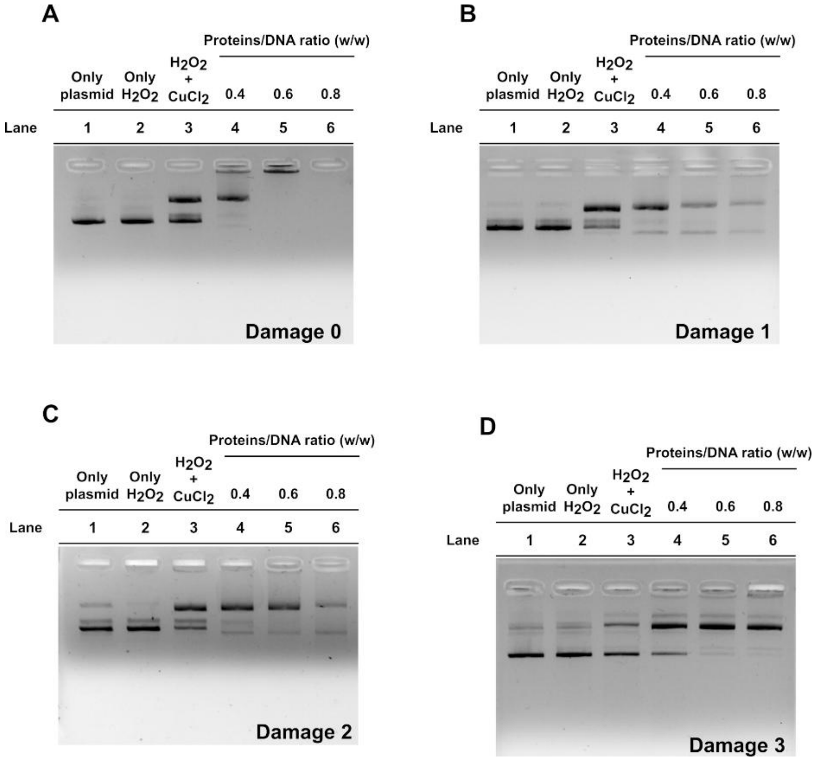

Supplementary Figure S2: Analysis on a 1% agarose gel of the ability of SNBPs to protect DNA damage of the pGEM3 plasmid in the presence of H_2O_2 and $CuCl_2$. The different damage groups are Grade 0 (A), Grade 1 (B), Grade 2 (C). The damage condition purposely created for plasmid DNA by adding 10 μM H_2O_2 and 5 μM $CuCl_2$ is shown in well 3 of all gels. The addition of SNBPs in the ratios 0.4 to 0.8 to DNA produces: no increase in damage, i.e., no increase in the intensity of the relaxed plasmid DNA band compared to the condition shown in well 3 revealing the ability of SNBP to aggregate DNA and protect from oxidative damage (Grade 0 (A)), a progressive decrease of relaxed DNA in the 0.4, 0.6 and 0.8 ratios, compared to the condition shown in well 3 (Grade 1 (B)), an increase in the intensity of the band corresponding to relaxed DNA at the 0.4, 0.6 ratio indicating that the SNBPs of these subjects have a low capacity to aggregate DNA (Grade 2 (C)); the conversion of all supercoiled DNA in relaxed form, indicating the involvement of the SNBP in the DNA oxidative damage. Supercoiled is the form of DNA plasmid ad higher mobility; relaxed is the form of DNA plasmid at lower mobility

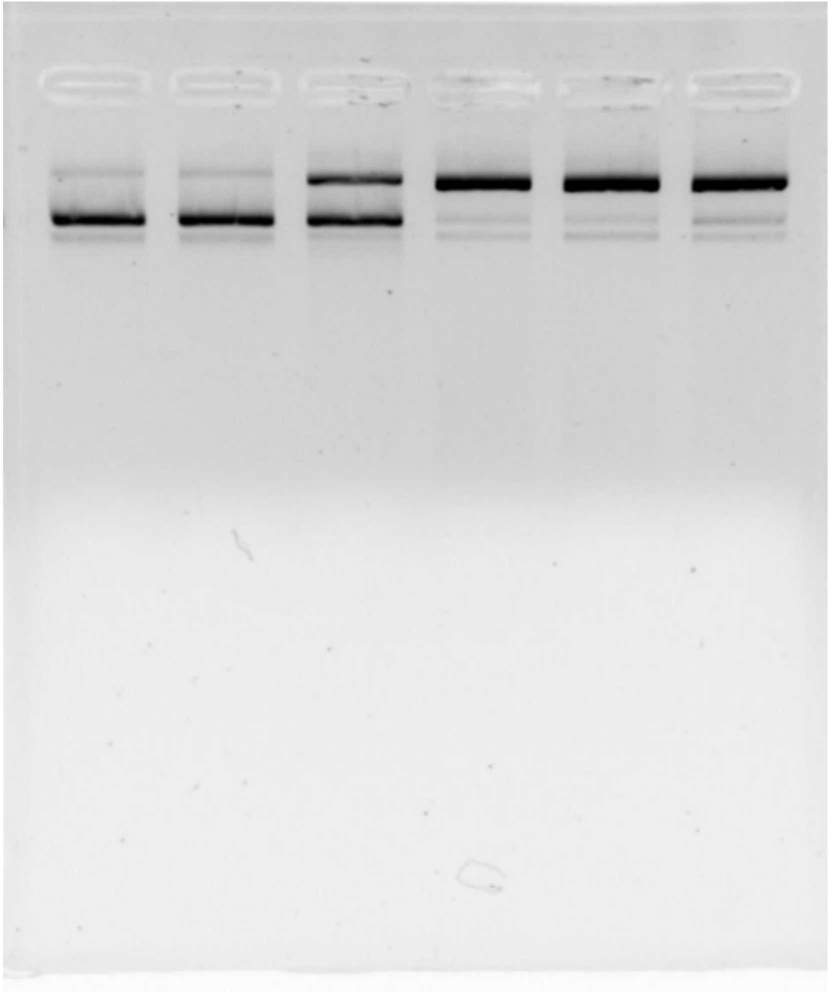

Supplementary Figure S3: Original image of Figure 4A.
